# Prospective surveillance of healthcare-associated infections and patterns of antimicrobial resistance of pathogens in an Italian intensive care unit

**DOI:** 10.1186/s13756-018-0337-x

**Published:** 2018-04-03

**Authors:** Aida Bianco, Maria Simona Capano, Valentina Mascaro, Claudia Pileggi, Maria Pavia

**Affiliations:** Department of Health Sciences, University of Catanzaro “Magna Græcia”, Via T. Campanella, 115, 88100 Catanzaro, Italy

**Keywords:** Healthcare-associated infection, Intensive care unit, Italy, Surveillance

## Abstract

**Background:**

The study aimed to evaluate the distribution of healthcare-associated infections (HAIs), the incidence rates and device utilization ratio (DUR) of device-associated infections (DAIs), as well as the distribution and patterns of antimicrobial resistance of the responsible pathogens.

**Methods:**

Eligible patients who were admitted to an adult Intensive Care Unit (ICU) from May 1, 2013 to December 31, 2016 were included in the surveillance. Demographics, intrinsic and extrinsic risk factors, information regarding infection and isolated pathogens with antibiogram results were collected.

**Results:**

One thousand two hundred eighty-three patients were included in the surveillance. One hundred forty-seven HAIs were detected with a cumulative incidence of 9.2 per 100 patients 4-year period and an incidence rate of 17.4 per 1000 patient days. Fifty-six out of 1283 patients were affected by at least one episode of ICU-acquired pneumonia, and 72.7% of these were associated with intubation. ICU-acquired bloodstream infections (BSIs) occurred in 4.4% of patients and 89.5% were catheter-related. ICU-acquired urinary tract infections (UTIs) occurred in 1% of patients, with 84.6% of the episodes being associated with the use of an urinary catheter. The pattern of antimicrobial-resistance in the isolates showed, among the Gram-positive bacteria, that 66.6% and 16.6% of *Staphylococcus epidermidis* were oxacillin and teicoplanin resistant, respectively. Among the Gram-negative bacteria, carbapenem resistance was found in 91.6% of *Acinetobacter baumannii* and 28.5% of *Klebsiella pneumoniae* isolates.

**Conclusions:**

The majority of HAIs in the ICU studied were associated with the use of invasive devices. Since a significant proportion of these HAIs are considered preventable, reinforcement of the evidence-based preventive procedures are needed.

**Electronic supplementary material:**

The online version of this article (10.1186/s13756-018-0337-x) contains supplementary material, which is available to authorized users.

## Background

Healthcare-associated infections (HAIs) represent a major threat to patient safety, leading to significant mortality and financial losses for health systems worldwide. In high-income countries, for every 100 hospitalized patients, 7 develop at least one HAI and the frequency of HAIs acquired by patients in intensive care units (ICUs) is at least 3 fold higher (approximately 30%) [1].

**Table 1:** Incidence of device-associated infections (DAIs) by site, 2013–2016

Year	VAP				CLABSI				CAUTI			
	*n*.	MVdays	Rate^a^	DUR^b^	*n*.	CLdays	Rate^a^	DUR^b^	*n*.	UCdays	Rate^a^	DUR^b^
2013	12	502	23.9	0.32	14	1241	11.2	0.81	0	1224	0^c^	0.80
2014	24	897	26.7	0.34	15	2255	6.6	0.86	2	2258	0.9	0.86
2015	10	915	10.9	0.40	10	1535	6.5	0.67	6	1560	3.8	0.68
2016	10	826	12.1	0.40	12	1528	7.8	0.75	3	1528	1.9	0.75
TOTAL	56	3140	73.6	1.46	51	6559	32.1	3.09	11	6570	6.6	3.09

NOTE. *VAP* ventilator-associated pneumonia, *CLABSI* central line-associated bloodstream infection, *CAUTI* catheter-associated urinary tract infection, *MV* mechanical ventilator , *DUR* device utilization ratio, *CL* central line, *UC* urinary catheter

^a^ $$ \mathrm{Device}-\mathrm{associated}\ \mathrm{infection}\ \mathrm{rate}=\frac{\mathrm{Number}\ \mathrm{of}\ \mathrm{device}-\mathrm{associated}\ \mathrm{infection}\mathrm{s}\ \mathrm{for}\ \mathrm{an}\ \mathrm{infection}\ \mathrm{site}}{\mathrm{Number}\ \mathrm{of}\ \mathrm{device}-\mathrm{days}}\mathrm{x}\ 1000 $$

^b^ $$ \mathrm{Device}\ \mathrm{utilization}\ \mathrm{ratio}\ \left(\mathrm{DUR}\right)=\frac{\mathrm{Number}\ \mathrm{of}\ \mathrm{device}-\mathrm{days}}{\mathrm{Number}\ \mathrm{of}\ \mathrm{patient}-\mathrm{days}} $$

^c^The rate was not calculated since the number of CAUTI in 2013 was 0

In Europe it is estimated that 4,131,000 patients are affected by HAI and approximately about 4,544,100 episodes occur each year [2]. HAIs cause 16 million extra days of hospital stay and 37,000 attributable deaths. The burden of HAIs in patients admitted to ICUs vary from 9.7–31.8% in Europe [3].

In Italy, a national network for the prospective surveillance of HAIs in all wards is not in place. Continuous multicenter infection surveillance systems have been adopted in ICUs, and reported cumulative incidence range from 9.1 [4] to 15.5 per 100 patients [5].

In the literature, variable proportions of HAIs, considered to be preventable by intensive hygiene and control programs, have been reported [6, 7]. Among the infection prevention initiatives, surveillance of HAIs is the cornerstone to decrease infection rates in hospitalized patients [8], and it is considered to be the best way to assure patient safety [9]. Continuous monitoring of HAI rates can be used to assess effectiveness of interventions and provides information which may be used for benchmarking comparison [10]. Routine surveillance of HAIs should become an integral part of infection prevention and quality assurance in hospitals [11].

This study reports the results of a 4-year period prospective surveillance in an ICU in an Italian teaching hospital aimed at evaluating: 1) the cumulative incidence and incidence rate of HAIs; 2) the incidence rates of device-associated infections (DAIs); 3) the device utilization ratio (DUR); 4) the distribution of the responsible pathogens; and 5) the patterns of their antimicrobial resistance.

## Methods

An HAIs surveillance system based on that of the Centers for Disease Control and Prevention’s (CDC) National Healthcare Safety Network (NHSN) [12] was established in 2013 by the Hospital Prevention & Control Group for the prospective surveillance of events and their corresponding denominator data by trained Infection Preventionists in an adult ICU.

### Setting and patients

The ICU is an 8-bed unit that is part of a university-affiliated teaching hospital, with two single rooms for isolation. The ICU type was classified according to the NHSN criteria as a medical/surgical unit of a major teaching hospital. It has approximately 382 admissions per year.

All eligible patients admitted from May 1, 2013 to December 31, 2016 have been included in the surveillance. Patients who were transferred to the ICU from an outside hospital are also included. The exclusion criteria were patients with a community acquired infection, ICU stay for less than 48 h and death within 48 h of ICU admission. The follow-up of each patient was continued until discharge, referral, or death.

### Data collection

Surveillance data on all ICU-acquired HAIs, both in patients with or without a device, and their causative pathogens were collected prospectively on a specifically designed form (Additional file 1) by the investigators using medical records comprising charts, daily flow sheets, laboratory (eg, complete blood count, serology, microbiology, biochemistry) and radiographic results. The collected data included demographics; intrinsic and extrinsic risk factors for infection; date of infection onset; clinical signs; administered antibiotics; isolated pathogens with antibiogram results; and outcome on discharge from the ICU. The risk factors were evaluated from the time of admission until the onset of HAI. For patients who did not develop HAI, the risk factors were evaluated for their entire ICU stay.

The definitions of HAIs, and DURs are based on those established by the CDC, which are used by the NHSN system [13]. Particular attention is paid to data on DAIs [ventilator-associated pneumonias (VAPs), central line–associated bloodstream infections (CLABSIs), and catheter-associated urinary tract infections (CAUTIs)], and denominator data (patient days and specific device days). An infection is defined as device-associated (i.e., mechanical ventilator (MV), urinary catheter (UC), or central line-associated (CL)) if the corresponding device was in place on the date of infection and within two calendar days prior. Surgical site infections were not monitored because we performed a ward-based surveillance, rather than a procedure-based surveillance.

Global prevalence of the pathogens causing DAIs and antimicrobial resistance rates are also calculated. Multidrug-resistance (MDR) is defined in accordance with current published interim standard definitions, which are used in the most recent NHSN antimicrobial resistance report [14].

### Statistical analysis

Rates and ratios are calculated according to standard methods [12]. Briefly, DUR are calculated as the total number of MV, CL, or UC days, divided by the total number of patient days. The infection frequency is calculated as the number of infections per 100 ICU eligible patients (cumulative incidence) and the number of infections per 1000 patient-days (incidence rate). Rates for DAIs are reported as the number of infections per 1000 device days.

The first part of the analysis examines the entire cohort of patients. Data are summarized using the mean and standard deviation (SD) for continuous variables and frequency and percentage for discrete and nominal variables. Univariate analysis was tentatively used to compare variables for the outcome groups of interest (HAIs versus no HAIs), and all tests of significance were two tailed. The T test was used for comparing continuous variables, while the χ^2^-statistic or Fisher-exact test was used for discrete and nominal variables. All statistical analyses were performed using the Stata software program, version 14 (Stata).

## Results

During the 4 year study period, 1283 patients were included in the surveillance. The population mean age was 66 (±12) years and 841 (66%) were males. Overall, 147 ICU-acquired HAIs were detected with a cumulative incidence of 9.2 per 100 patients 4-year period and an incidence rate of 17.4 per 1000 patient days. A total of 118 DAIs were found, of which 38.1% were VAPs, 34.7% CLABSIs and 7.5% CAUTIs.

In the population, 56 out of 1283 patients (6%) were affected by at least one episode of ICU-acquired pneumonia, and 72.7% of these were VAPs. The incidence rate of ICU-acquired pneumonia was 9 episodes per 1000 patient-days and VAP incidence rate was 17.8 per 1000 MV days (> 90^th^ percentile).

On average, ICU-acquired BSIs occurred in 4.4% of patients staying in an ICU for more than 2 days. The incidence rate was 6.8 BSI episodes per 1000 patient-days. 89.5% of cases were CLABSIs with an incidence rate of 7.7 per 1000CL days (> 90^th^percentile).

On average, ICU-acquired urinary tract infections (UTIs) occurred in 1% of patients staying in an ICU for more than 2 days, with 84.6% of UTI episodes being associated with the use of a UC The incidence rate per ICU was 1.5 UTI episodes per 1000 patient-days and a mean device-adjusted rate of 1.6 CAUTI episodes per 1000 UC -days (25^th^- 50^th^ percentile).

The trend during the surveillance period showed that the rate of VAP considerably decreased from 23.9 in 2013 to 12.1 in 2016 per 1000 MV days; CLABSI reduced from 11.2 in 2013 to 7.8 in 2016 per 1000 CL days. The incidence rate of CAUTI had been constantly contained during the surveillance period, ranging from 0 in 2013 to 3.8 in 2015, while in 2016 it was found to be 1.9 per 1000 UC days (Table 1).

Among Gram-negative bacteria, the most common isolated pathogens were *Klebsiella pneumoniae* (17.8%), *Acinetobacter baumannii* (10.2%), *Escherichia coli* (8.5%), *Pseudomonas aeruginosa* (5%); among Gram-positive bacteria *Staphylococcus epidermidis* (10%) was the most common. All yeasts found were classified as *Candida species.* Throughout the surveillance period, *Klebsiella pneumoniae* was the most frequent pathogen associated with VAPs (*n* = 12) followed by *Acinetobacter baumannii* (*n* = 6), *Escherichia coli* (*n* = 6) and *Pseudomonas aeruginosa* (*n* = 6). Thirteen VAP episodes were non-microbiologically confirmed. The most frequently isolated microorganisms in ICU-acquired CLABSI episodes were coagulase-negative staphylococci (*n* = 21) and of these 11 were *Staphylococcus epidermidis*; among Gram-negative bacteria *Klebsiella pneumoniae* (*n* = 7) and *Acinetobacter baumannii* (*n* = 5) were the most frequent isolates. *Candida species* (45.4%) and *Klebsiella pneumoniae* (18.1%) were the most frequently isolated microorganisms in CAUTI episodes.

The antimicrobial-resistance in the isolates associated with ICU-acquired DAIs showed, among the Gram-positive bacteria, that 66.6% and 16.6% of *Staphylococcus epidermidis* isolates were β-lactam (oxacillin) - and glycopeptide (teicoplanin) - resistant, respectively. Among the Gram-negative bacteria third-generation cephalosporins (cefotaxime or ceftazidime) resistance was found in 52.3% of *Klebsiella pneumoniae* and 30% of *Escherichia coli* isolates; and carbapenem (imipenem, meropenem, ertapenem) resistance in 91.6% of *Acinetobacter baumannii* and 28.5% of *Klebsiella pneumoniae* isolates. MDR phenotypes were reported in all *Pseudomonas aeruginosa,* in *Acinetobacter baumannii* (91.6%), *Escherichia coli* (40%) and *Klebsiella pneumoniae* (52.3%) isolates. Resistance to colistin, an antibiotic from the polymyxin group, was observed in 4% of the *Klebsiella pneumoniae* isolates.

Results of univariate analysis showed that no statistically significant difference between infection and the independent covariates was found (data not shown).

## Discussion

The cumulative incidence of HAIs in ICU wards varies widely among countries (5%–38.9%) [15], and the detected rate in the present surveillance (9.2%) was in the range of values specified in other studies. Data reported by The European Surveillance System showed that, in 2014, 8% of the patients staying in the ICU for more than 2 days presented at least one HAI [16]. The HAI incidence rate of 17.4 per 1000 patient days was slightly higher than that noticed in the study from the USA where the rate of HAI in an adult ICU was shown to be 16.2 per 1000 patient days[17]. The majority of HAIs in ICUs are associated with the use of invasive devices. Therefore, our surveillance system paid particular attention to DAIs, since a significant proportion of these HAIs are considered preventable. The findings showed that the most common DAI was VAP (47.5%), consistent with the reported literature. Globally, the VAP rate we observed (17.8/1000 MV days) was higher than the surveillance data from Germany (5.4/1000 MV days) [18] and from the United States where the VAP incidence rate was 2.1 per 1000 device days in medical/surgical major teaching ICUs [19]. A more pertinent benchmarking information could be obtained on a national scale. The cumulative incidence of the ICU-acquired HAIs (9.2%) is very similar to that reported in a continuous multicenter infection surveillance program (9.1%) involving 125 Italian ICUs promoted by the Italian Group for the Evaluation of Interventions in Intensive Care Medicine [4]. Similarly, VAP was the most frequently diagnosed, but the incidence rate was lower than that reported in the present surveillance program (8.9/1000 vs 17.8/1000 MV days) [4]. However, the Italian Nosocomial Infections Surveillance in ICUs network, SPIN-UTI, reported surveillance data from a 6-year period (2006–2011), with a third survey showing a more similar VAP incidence rate (17.3 per 1000 MV days) [20], while the VAP incidence rate was higher in a surveillance program organized in an Italian single-centre 12-bed ICU (23.14/1000 MV days) [21].

The CLABSI incidence rate (7.7 per 1000 CL days) we observed was much lower than that reported from limited-resource countries [22, 23], but higher than 1.1 per 1000 CL days reported in the medical/surgical major teaching ICUs in the healthcare facilities adhering to NHSN surveillance [19]. In the SPIN-UTI, in the 2010–2011 survey, and in the GiViTi projects the CLABSI incidence rate was 1.8 and 1.9 per 1000 CVC days, respectively [20, 4]. The higher rate in the present surveillance could be explained by a high CL utilization ratio, exceeding the 90th percentile of the NHSN distribution for medical/surgical major teaching ICUs. Intravascular devices remain an essential component of ICU care, but many studies recognize CL duration as a risk factor for CLABSI [24, 25].

The reasons for lower CAUTI rates observed in the present study compared with other studies may be related to the effectiveness of the interventions implemented in our setting. They included educational strategies, UC avoidance, policies for UC insertion, daily necessity review and limiting catheter days that have been proven to decrease CAUTI events [26].

In the present study Gram-negative bacteria were the most common causal pathogens, in agreement with several surveillance studies in the United States [27], Europe [28], Saudi Arabia [29] and Brazil [30]. Among these Gram-negative bacteria, *Klebsiella pneumoniae* (17.8%) and *Acinetobacter baumannii* (10.2%) were the most frequently reported. This finding is of particular concern, since these organisms are often involved in outbreaks that require the activation of an organizational response until the outbreak is under control [31].

In the present study, the high level of resistance to multiple antibiotics is of great concern, especially since resistance to colistin was also reported. This condition represents an indication of seriously limited options for the treatment of patients infected with those microorganisms.

A variability in DAI rates across the surveillance period can be observed. Part of this variability can be attributed to the small number of episodes, and characteristics of the patient population, such as clinical severity and infection control practices that had been reinforced during surveillance. Over the 4 year study several infection control practices, such as the active surveillance cultures to detect asymptomatic colonization and preemptive contact isolation of patients were implemented.

The strengths of the study lie in the prospective nature, and application of NHSN methodology. It is well known that indicators of HAIs provided by surveillance activities require comparison with adequate reference data to stimulate further infection control actions and to enhance quality of care [32]. The NHSN methodology has proven to be adaptable in our context and useful as benchmark.

This study had a couple of limitations. An HAI incidence study should preferably be performed on large cohorts, taken from a national ICU sample. The consequence of a small population size is the low probability of detecting an important effect, since the analysis is underpowered. For this reason, we did not show the results of the univariate analysis. The present surveillance was conducted in one adult ICU in Southern Italy, and the results cannot be generalized to all public Italian hospital settings. It is reasonable to suppose that an analogous context may be referred to the Southern part of our country.

## Conclusion

Given the limitations cited above, the authors believe that to have more insight into HAI indicators as measured by a surveillance system, it is crucial to estimate the burden of HAIs and to compare the performance to that of other contexts, as well as to evaluate new preventive procedures.

## Additional file

Additional file 1: Data Collection Form. (DOC 330 kb)
