# Supplementary material for: Prospective surveillance of healthcare-associated infections and patterns of antimicrobial resistance of pathogens in an Italian intensive care unit
Source: Antimicrob Resist Infect Control. 2018 Apr 3;7:48. doi: 10.1186/s13756-018-0337-x (PMC5883356; doi:10.1186/s13756-018-0337-x)
Supplement: Supplementary file 1 — Data Collection Form. (DOC 330 kb) [file 13756_2018_337_MOESM1_ESM.doc]

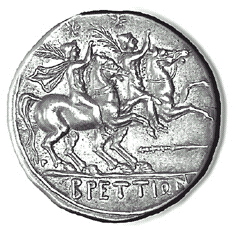
DEPARTMENT OF HEALTH SCIENCES

MEDICAL SCHOOL

UNIVERSITY OF CATANZARO “MAGNA GRÆCIA”

Prospective surveillance of healthcare-associated infections and patterns of antimicrobial resistance of pathogens in an Italian Intensive Care Unit

*Data Collection Form*

Survey date: ___/ ___/ ______; ___/ ___/ ______; ___/ ___/ ______; ___/ ___/ ______; ___/ ___/ ______

# 1. PATIENT DATA

ID Number: _____ Medical record number: _____ Gender: ❑ Male ❑ Female Date of birth: ___/ ___/ ____

Diagnosis of admission: _______________________________________________________________________

Admit source: ❑ Community ❑ Other hospital (_________________________________________) ❑ E.R.

Admission date: ____/ _____/ ______ Admit unit: ❑ ICU ❑ Other: ___________________

Discharge date: ____/ _____/ ______ Diagnosis at discharge: ___________________________________

Status: ❑ Ordinary discharge ❑ Transferred ❑ Deceased

2. INTRISIC RISK FACTORS

❑ Alcohol ❑ Diabetes ❑ Cirrhosis ❑ Chronic lung disease ❑ Coma ❑ Smoke ❑ Liver disease ❑ Obesity

❑ Hypertension ❑ Malignancy ❑ Chronic kidney disease

❑ Other_______________________________________________________________________________

3. EXTRINSIC RISK FACTORS

❑ Mechanical ventilation Start date ____/ ____/ _____ End date ____/ ____/ ______

❑ Central vascular catheter Site_________________________________________________

Start date ____/ ____/ _____ End date ____/ ____/ ______

❑ Peripheral vascular catheter Site_________________________________________________

Start date ____/ ____/ _____ End date ____/ ____/ ______

❑ Urinary catheter Start date ____/ ____/ _____ End date ____/ ____/ ______

❑ Nasogastric tube Start date ____/ ____/ _____ End date ____/ ____/ ______

❑ Drainage device Start date ____/ ____/ _____ End date ____/ ____/ ______

❑ Counter pulsation Start date ____/ ____/ _____ End date ____/ ____/ ______

## 4. ANTIMICROBIAL THERAPY

## ❑ No ❑ Yes Antimicrobial molecule: ______________________________________________ Start date: ____/ ____/ ______ End date: ____/ ____/ ______

## ❑ No ❑ Yes Antimicrobial molecule: ______________________________________________ Start date: ____/ ____/ ______ End date: ____/ ____/ ______

## ❑ No ❑ Yes Antimicrobial molecule: ______________________________________________ Start date: ____/ ____/ ______ End date: ____/ ____/ ______

## 6. HEALTHCARE - ASSOCIATED INFECTION

| HAI 1 | HAI 2 | HAI 3 |
| --- | --- | --- |
| ❑ No ❑ Yes | ❑ No ❑ Yes | ❑ No ❑ Yes |
| Onset date  ___/ ___/ _____ | Onset date  ___/ ___/ _____ | Onset date  ___/ ___/ _____ |
| Site  ❑PNEU ❑VAP  ❑BSI ❑CLABSI  ❑UTI ❑CAUTI | Site  ❑PNEU ❑VAP  ❑BSI ❑CLABSI  ❑UTI ❑CAUTI | Site  ❑PNEU ❑VAP  ❑BSI ❑CLABSI  ❑UTI ❑CAUTI |

7. CLINICAL SIGNS AND SYMPTOMS

❑ Fever ≥ 38ºC ❑ Hypotension (BP < 90/60) ❑ Dyspnea ❑ Tachypnea (RR > 25) ❑ Tachycardia (HR > 100)

❑ Nausea/Vomiting ❑ Dysuria ❑ Suprapubic Tenderness ❑ Urinary urgency ❑Other_______________

1. Chest X rays: Date ____/ ____/ ______ Result: _____________________________________

2. Chest X rays: Date ____/ ____/ ______ Result: _____________________________________

3 .Chest X rays: Date ____/ ____/ ______ Result: _____________________________________

8. MICROBIOLOGY

| Specimen Source  (e.g., blood, urine) | Date | Result | Antibiotic Resistance |
| --- | --- | --- | --- |
|  |  |  |  |
|  |  |  |  |
|  |  |  |  |
|  |  |  |  |
|  |  |  |  |
|  |  |  |  |
|  |  |  |  |
|  |  |  |  |
|  |  |  |  |
|  |  |  |  |
